# Vascularized omental lymphatic transplant for upper extremity lymphedema: A systematic review

**DOI:** 10.1002/cnr2.1370

**Published:** 2021-04-07

**Authors:** Nicholas R. Jarvis, Ricardo A. Torres, Francisco R. Avila, Antonio J. Forte, Alanna M. Rebecca, Chad M. Teven

**Affiliations:** ^1^ Mayo Clinic Alix School of Medicine Mayo Clinic Scottsdale Arizona USA; ^2^ Division of Plastic Surgery Mayo Clinic Jacksonville Florida USA; ^3^ Division of Plastic and Reconstructive Surgery, Department of Surgery Mayo Clinic Phoenix Arizona USA

**Keywords:** lymphedema, microsurgery, omental flap, upper extremity, vascularized omental lymphatic transplant

## Abstract

**Background:**

Vascularized omental lymphatic transplant (VOLT) is an increasingly popular treatment of extremity lymphedema given its promising donor site. While the success of VOLT in the treatment of lymphedema has been reported previously, several questions remain.

**Aim:**

To further elucidate appropriate use of VOLT in the treatment of lymphedema, specifically addressing patient selection, harvest technique, and operative methods.

**Methods and Results:**

A systematic review of VOLT for upper extremity lymphedema was performed. Of 115 yield studies, seven were included for analysis based on inclusion and exclusion criteria. Included studies demonstrated significant reductions in extremity circumference/volume (average volume reduction, 22.7%‐39.5%) as well as subjective improvements using patient‐reported outcomes. Though studies are heterogenous and limited, when analyzed in aggregate, suggest the efficacy of VOLT in lymphedema treatment.

**Conclusion:**

This is the largest systematic review of VOLT to date. VOLT continues to show promise as a safe and efficacious surgical intervention for lymphedema in the upper extremity. Further studies are warranted to more definitively identify patients for whom this technique is appropriate as well as ideal harvest and inset technique.

## 1 INTRODUCTION

**FIGURE 1:**
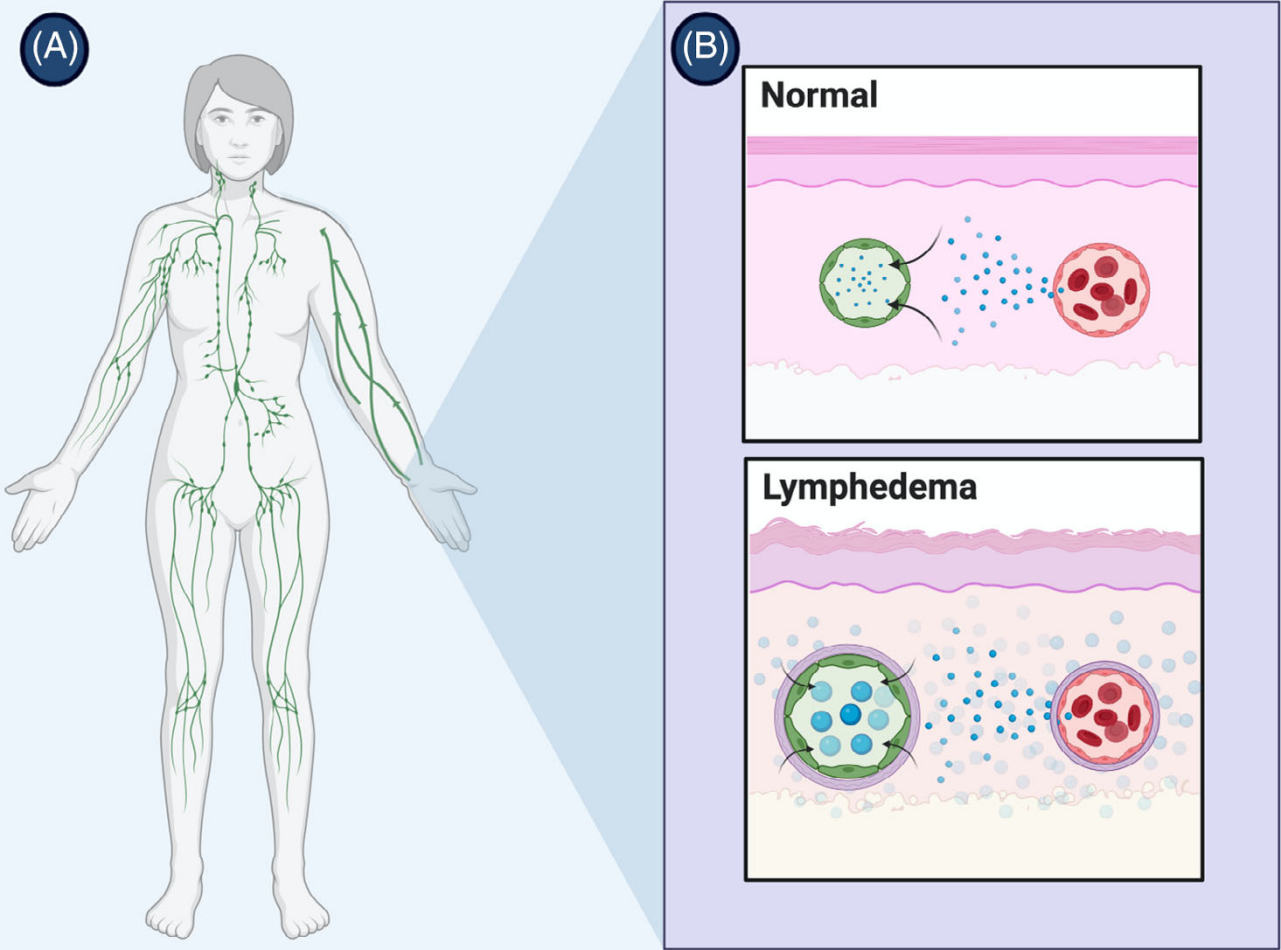
A, Tissue swelling due to lymphedema. B, The lymphatic system's capacity is exceeded, causing accumulation of fluid in the interstitium, which promotes the deposition of collagen and the proliferation of adipocytes around the capillary and collecting lymphatics

Lymphedema is a progressively debilitating disease process that results from the accumulation of protein‐rich interstitial fluid from insufficient or impaired lymphatic system function (Figure 1).^1^, ^2^, ^3^ Further characterization is defined by the etiology of the disease, with primary lymphedema being a result of congenital abnormalities in the lymphatic system and secondary lymphedema being a result of injury, disease, or iatrogenic processes causing lymphatic dysfunction.^2^ In developed countries, the most common cause of lymphedema is cancer and related treatment (eg, radiation, lymph node dissection).^4^ The development of this disease often leads to functional impairment, pain and discomfort, and diminishes quality of life, thus underscoring the importance of effective interventions to restore lymphatic function.^5^ Upper extremity lymphedema is a common sequela of breast cancer treatment, due to frequent axillary lymph node irradiation and dissection.^6^ The functional and psychosocial morbidities associated with upper extremity lymphedema in this cohort have proven to be disabling, and warrant review of the most effective treatments.^7^

Conservative approaches to lymphedema management—including compression garments, manual lymphatic drainage, and complex decongestive physiotherapy—vary widely in accessibility and efficacy.^8^, ^9^ If these measures fail, a variety of surgical interventions are available, most of which exist under the categories of excisional or physiologic techniques.^2^ The two commonest physiologic microsurgical techniques include lymphovenous bypass (LVB) and vascularized lymph node transplant (VLNT). VLNT in particular has gained recent traction. This procedure utilizes the transfer of healthy vascularized lymphatic tissue to improve lymphatic drainage in affected areas.^10^ The preferred donor site varies by patient, with inguinal, supraclavicular, and submental nodes commonly utilized. However, these sites are associated with significant risks, including donor‐site lymphedema, lymphocele, and unsightly scarring.^11^, ^12^

**FIGURE 2:**
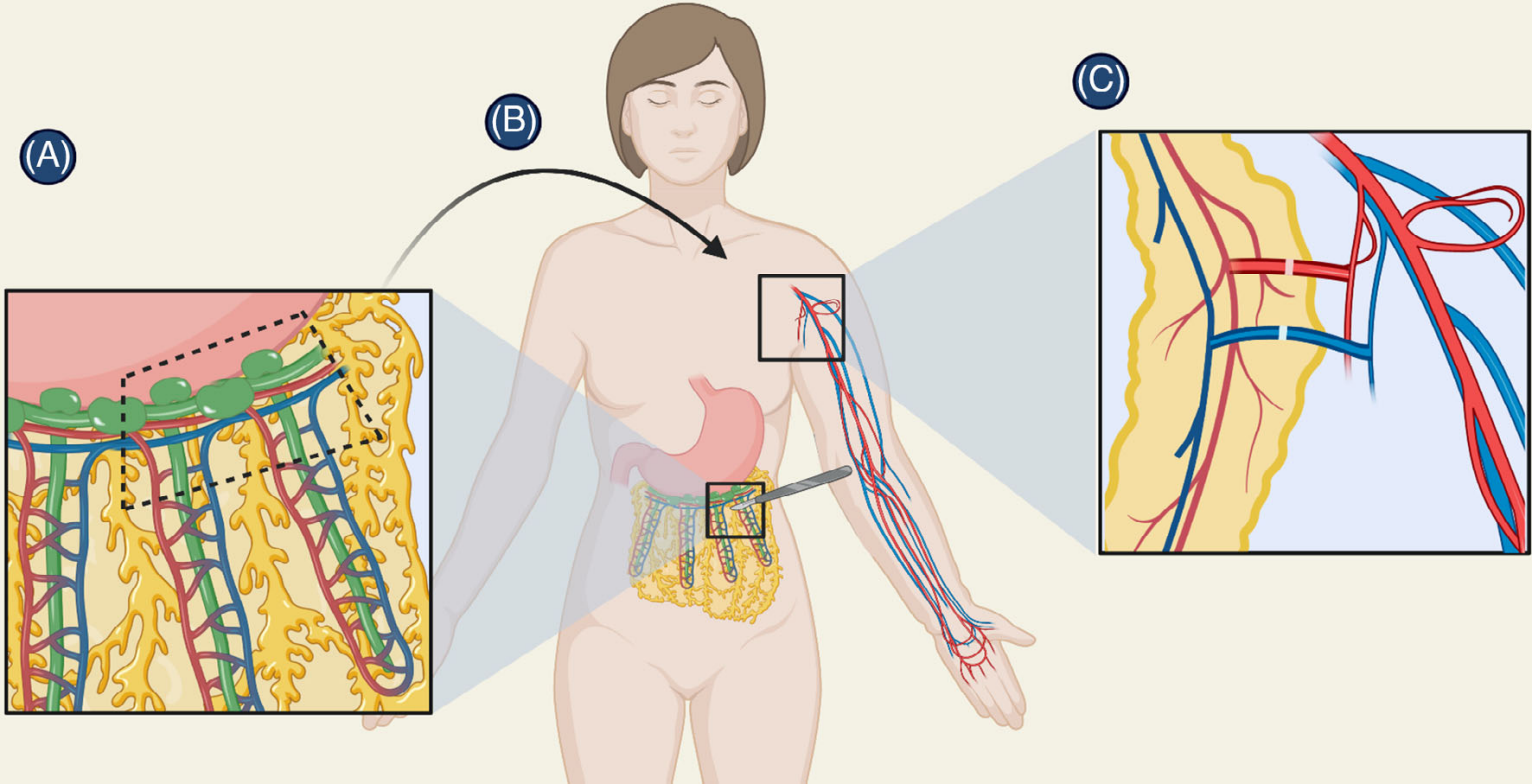
A, Healthy nodes en bloc and blood vessels from the omentum are B, transferred to the recipient site. C, Using microsurgery techniques, the blood vessels are anastomosed to the recipient site

More recently, use of the omentum has increased in popularity due in large part to its improved complication profile, particularly with respect to donor‐site lymphedema (Figure 2). Traditionally, the omentum is harvested via laparotomy. Recent advances have allowed for minimally invasive harvesting techniques, including laparoscopically.^13^, ^14^ Initially utilized for coverage of complex defects (eg, sternal wound coverage), the omentum has also been recently utilized in the treatment of lymphedema. The omentum carries many advantages—abundance of lymphatic tissue, immunogenic properties, lymphangiogenic properties—which have spurred the increased use of vascularized omental lymphatic transplant (VOLT), also referred to as gastroepiploic vascularized lymph node transfer (GE VLNT), as a surgical treatment option for refractory lymphedema.^15^, ^16^

While the success of VOLT in the treatment of lymphedema has been reported previously, several questions remain.^3^ For example, appropriate application of VOLT, proper patient selection, and harvest technique have not yet been clearly identified. Further, the specific role of VOLT in upper extremity lymphedema is of interest. Additionally, the number of authors evaluating surgical treatments for lymphedema is increasing at a fast pace in light of recently published positive findings. Thus, it is important to update our knowledge base on a frequent basis. Therefore, the purpose of this systematic review is to incorporate previously reported data with more recently reported findings on the use of VOLT in UE lymphedema and its associated outcomes. Because physiologic approaches to lymphedema management such as VOLT are newer and less well studied than older extirpative techniques such as direct excision and liposuction, it is paramount that we perform ongoing and regularly updated analyses of such techniques. This helps to ensure that novel therapies are safe, effective, and appropriate. Further, it provides increased information regarding optimal patient selection, choice of operation, and technical details. Finally, we hope to provide readers, who may or may not have experience with lymphedema treatment but nevertheless may encounter in their practice patients at risk for or with lymphedema, with knowledge and context to facilitate effective counseling.

## 2 METHODS

### 2.1 Literature review

A systematic literature review of electronically available publications was performed on June 19, 2020. Two reviewers (NJR and CMT) performed the searches in independent fashion without timeline limitations. A third review (AMR) resolved any disagreements regarding article identification and inclusion/exclusion as noted below. The data that support the findings of this study are available from the corresponding author upon reasonable request. The review was conducted in accordance with Preferred Reporting Items for Systemic Reviews and Meta‐Analyses (PRISMA) guidelines.

### 2.2 Search criteria

An all language search of four online databases (Medline, Embase, Web of Science, and Scopus) was performed using the following keywords: lymphedema AND vascularized omental lymphatic transplant OR VOLT OR lymph node transfer OR lymph node transplant OR lymph node flap OR lymphatic transplant AND vascularized AND omentum OR omental OR gastroepiploic AND upper extremity OR upper limb OR shoulder OR arm OR forearm OR elbow OR wrist OR hand OR finger.

### 2.3 Inclusion criteria

All studies returned from the search using the described keywords were reviewed for inclusion. Two authors independently reviewed the results to ensure appropriate inclusion of studies that utilized VOLT as a treatment for upper extremity lymphedema, including all subtypes. Disagreements regarding article identification and selection for inclusion were resolved by a third reviewer.

### 2.4 Exclusion criteria

**FIGURE 3:**
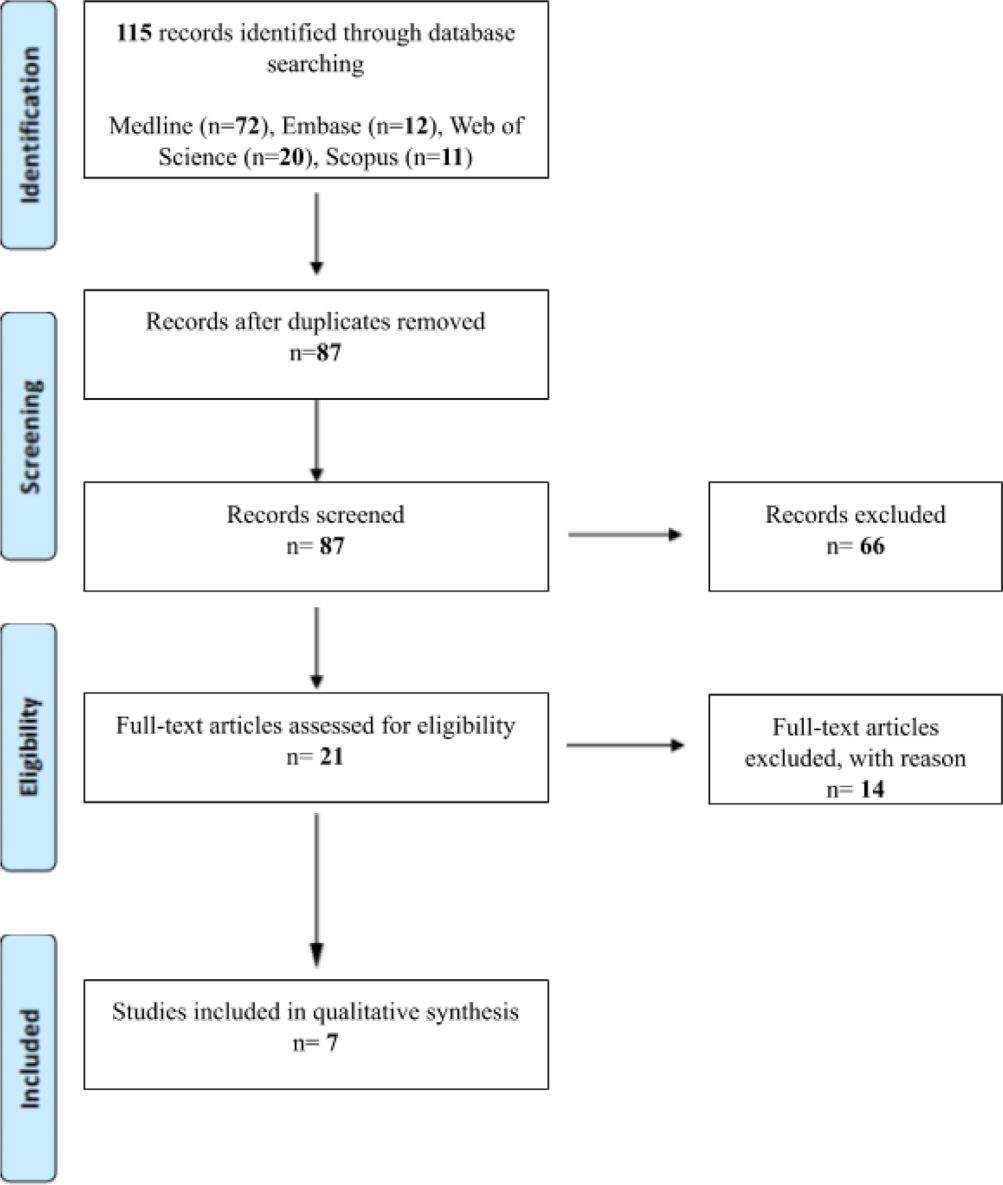
PRISMA Flowchart depicting search schema (Preferred Reporting Items for Systematic Reviews and Meta‐Analyses)

A title/abstract screening was performed to remove any results not pertaining to VOLT as a treatment for upper extremity edema. We excluded papers that did not report VOLT as a lymphedema treatment and also those that were singularly focused on lower extremity treatment. Duplicates, abstracts, presentations, systematic reviews, meta‐analyses, case reports, nonclinical studies, studies without descriptive outcomes, and non‐English studies were additionally excluded. The search schema and review methodology are described using a PRISMA diagram in Figure 3.

### 2.5 Data extraction and synthesis

Extracted data included author group, year of study, and country of origin; demographic data including patient age and population; lymphedema etiology and stage; surgical intervention including omental harvest technique (ie, open vs laparoscopic vs robotic); lymphoscintigraphy; clinical outcome (subjective and objective when provided); and duration of follow‐up and complications. Two reviewers (NRJ and CMT) performed data extraction from articles, figures, and tables. Accuracy of entered data was confirmed by a third review (AMR). Data was synthesized and reported in the results section as noted below.

## 3 RESULTS

The initial literature search yielded a total of 115 articles, with 87 originals and 28 duplicates. Following initial title/abstract review, 66 articles not pertaining to VOLT as a treatment for UE lymphedema were excluded. The remaining 21 articles underwent full‐text review, with 14 articles being excluded due to being case reports, studies without descriptive outcomes, or systematic reviews. The remaining seven articles satisfied all inclusion and exclusion criteria and were included in the review below. Of the seven studies included, three were prospectively designed, while four were retrospective cohort studies.

Seven studies, which were published between 2017 and 2020, fulfilled inclusion criteria for review, with the total number of patients included being 91 with ages ranging from 27 to 72 (Table 1).^17^, ^18^, ^19^, ^20^, ^21^, ^22^, ^23^ All but one patient were female and all patients experienced secondary lymphedema, with the majority being related to breast cancer. Forty‐five patients were diagnosed with Stage II lymphedema and 14 patients were diagnosed with Stage III lymphedema, according to the International Society of Lymphology (ISL) staging criteria. One study of 32 patients did not include ISL staging.^23^ All studies used variations of VOLT as an intervention for upper extremity lymphedema (Table 2). Variations of this procedure included single VOLT,^18^, ^20^, ^21^, ^23^ double VOLT,^17^, ^22^, ^23^ and double VOLT with suction‐assisted lipectomy (SAL).^19^ Further variation existed in the use of middle or distal upper limb inset, both within and between studies. Two studies noted the use of laparotomy, rather than laparoscopy, for lymph node harvest.^20^, ^23^ All patients experienced a reduction in upper extremity circumference or volume within the follow‐up range of 0.5 to 4 years. Between studies, the average circumference reduction ranged from 37.8% to 74.5% and the average volume reduction ranged from 22.7% to 39.5%. Of the studies that reported data on cellulitis, there was a significant reduction or complete absence of cellulitis episodes postoperatively.^20^, ^22^, ^23^ Lymphoscintigraphy was commonly used pre‐ and postoperatively and demonstrated significant improvement in lymphatic drainage.^17^, ^18^, ^21^, ^22^, ^23^ Several studies noted improvements in patient‐reported outcomes, such as quality of life, function, appearance, and symptoms, although there was heterogeneity among measurement modalities.^17^, ^18^, ^20^, ^21^, ^23^ Complications included sensory abnormalities (5.5%),^17^, ^19^ partial skin graft loss (4.4%),^18^, ^19^ vascular compromise of a flap (2.2%),^20^, ^23^ ileus (2.2%),^23^ flap loss (1.1%),^23^ transient pancreatitis (1.1%),^23^ and infection (1.1%).^19^ There were no reports of donor site lymphedema.

**TABLE 1:** Patient characteristics of included studies on the use of vascularized omental lymphatic transplant for upper extremity lymphedema

Author	Demographic data	Age mean (range)	Lymphedema etiology	ISL stage
Ciudad et al^17^ 2019 Taiwan	6 patients Female: 6	57.8 years old (47‐65)	Breast‐cancer related: 6	Stage III: 6
Manrique et al^18^ 2020 USA	14 patients Female: 13 Male: 1	51.8 years old	Secondary:14	Stage II: 14
Agko et al^19^ 2018 Taiwan	6 patients Female: 6	52 years old	Breast cancer‐related: 6	Stage II: 6
Mousavi et al^20^ 2020 Iran	24 patients Female: 24	48.7 years old (35‐70)	Breast‐cancer related: 24	Stage II: 24
Ciudad et al^21^ 2017 Taiwan	5 patients Female: 5	52.4 years old (48‐60)	Breast‐cancer related: 5	Stage II: 1 Stage III: 4
Ciudad et al^22^ 2017 Taiwan	4 patients Female: 4	53 years old (42‐62)	Breast‐cancer related: 4	Stage III: 4
Kenworthy et al^23^ 2018 USA	32 patients Female: 32	54.9 years old (27‐72)	Breast‐cancer related: 30	N/A

**TABLE 2:** Procedure descriptions and outcomes of included studies on the use of vascularized omental lymphatic transplant for upper extremity lymphedema

Author	Follow‐up Mean (range)	Intervention	Omental Harvest Technique	Clinical Outcome	Lymphoscintigraphy	Complications
Ciudad et al^17^ 2019 Taiwan	14.8 months (12‐19)	Combined double GE VLNT and modified RRPP	Laparoscopy	Mean circumference reduction: 74.5% Improvement in quality of life, function, appearance, and symptoms	1‐year postoperative: significant improvement in lymphatic drainage when compared to preoperative imaging	Paresthesia: 1 Hyperesthesia: 1
Manrique et al^18^ 2020 USA	7.3 months	GE VLNT	Laparoscopy	Excess volume reduction: 22.7% Significant improvement in physical symptoms, psychosocial, and functional outcomes	1‐year postoperative: new focal uptake, improved radiotracer transit time, and greater avidity of tracer	Partial Skin Graft Loss: 3
Agko et al^19^ 2018 Taiwan	Performed at 2 weeks, 1 month, 3 months, 6 months, and every 3 months thereafter	Staged dual GE VLNT with suction‐assisted lipectomy (SAL)	Laparoscopy	Overall circumference reduction rate: 37.8% post VOLT; 97.7% post VOLT + SAL	NS	Transient numbness: 3 Infection: 1 Partial skin graft loss: 1
Mousavi et al^20^ 2020 Iran	(1–4 years)	GE VLNT	Laparoscopy and laparotomy	Significant reduction of upper extremity circumferential size Significant reduction in number of annual cellulitis episodes (7 to 0.3) Significant improvement, satisfaction, function, and appearance	NS	Flap venous compromise: 1
Ciudad et al^21^ 2017 Taiwan	14.4 months (13‐18)	GE VLNT	Laparoscopy	Mean volume reduction: 39.5% 2.6‐fold improvement in quality of life	Perioperative: transplanted lymph node viability and improved lymphatic transport	None
Ciudad et al^22^ 2017 Taiwan	9.25 months (8‐11)	Double GE VLNT	Laparoscopy	Mean circumference reduction rate: 41.5% No episodes of cellulitis	6‐month postoperative: significant improvement in lymphatic drainage	None
Kenworthy et al^23^ 2018 USA	9.7 months (0.5‐24)	Double VOLT: 12 patients Single VOLT: 20 patients	Laparotomy	Observed clinical improvement Reduction in cellulitis episodes from 44.7% to 13.2%	1‐year postoperative: physiologic function in 50% of double VOLT patients and 56% of the overall cohort	Flap loss: 1 Arterial anastomosis avulsion: 1 Transient pancreatitis: 1 Ileus: 2

Abbreviations: GE, Gastroepiploic; RRPP, radical reduction with preservation of perforators; VLNT, Vascularize Lymph Node Transfer.

## 4 DISCUSSION

Historically, LVB has been the preferred technique for the microsurgical treatment of lymphedema. LVB involves anastomosing a lymphatic vessel to a recipient vein with the goal of bypassing the proximal obstruction within the lymphatic system and thus improving lymphatic outflow.^24^ However, its efficacy in later stages of lymphedema has been brought into question.^24^, ^25^ While recent use of VLNT as an alternative has shown promise, the ideal donor site has yet to be determined. Important considerations in this determination include: risk of iatrogenic lymphedema, nerve injury, scar appearance, and abundance of lymph nodes at the donor site.^19^ The combination of recent advancements in laparoscopic technique and the advantageous lymphatics and vasculature of the omentum have made it a safe alternative to the more commonly used inguinal, supraclavicular, submental, and lateral thoracic sites.^3^, ^15^, ^16^ However, intra‐abdominal harvest comes with risks of visceral injury and incisional hernia.^26^ Therefore, donor site selection must account for the potential benefits and risks of each site.

**FIGURE 4:**
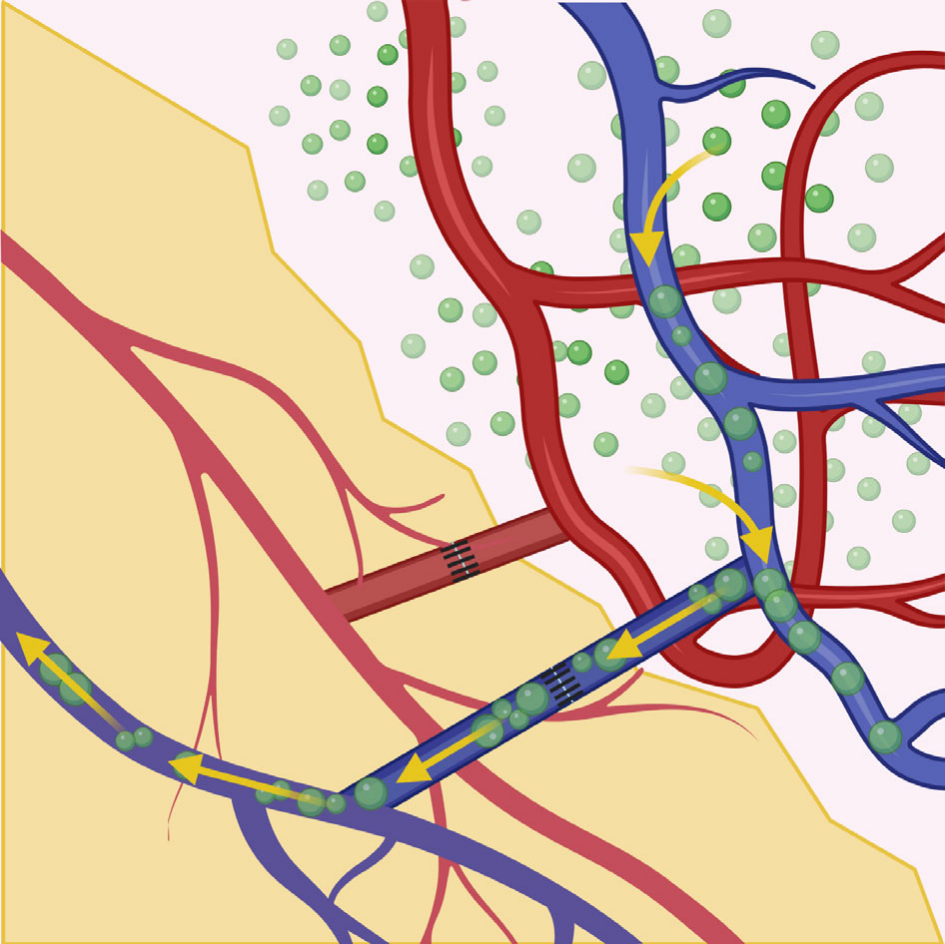
The vascularized omentum transfer acts as a “pump,” draining the fluid trapped in the interstitium to the venous system through a connection between the flap and the patient's recipient vein, alleviating the swelling

The results of this systematic review, which to our knowledge is the first to specifically address VOLT for UE lymphedema, support the use of VOLT as an effective treatment for upper extremity lymphedema. The vascularized omentum transfer acts as a “pump,” draining the fluid trapped in the interstitium to the venous system through a connection between the flap and the patient's recipient vein, alleviating the swelling (Figure 4). While positive outcomes were common among all the studies included herein, there was considerable variation in the technique used by the authors, with some using single VOLT (single‐level inset) or double VOLT (double‐level inset from single flap). Variation also exists in the recipient site, with the antecubital fossa (middle inset) and volar wrist (distal inset) being common sites in these studies. Manrique et al compared outcomes between these two sites and found that while there was no significant difference between volume reduction and functional improvements, patients with the middle inset had significantly shorter hospital stays and higher satisfaction with scar appearance, often due to the necessity of a skin graft for the distal inset.^18^ The axilla (proximal inset) is another site that is commonly used for flap inset. However, Montag et al found no difference in volume reduction between the proximal and distal insets.^27^, ^28^ Proximal inset is advantageous in that it allows for the release of postsurgical/radiation scar tissue within the axilla, and also potentially allow for decompression of the axillary vein. However, the presence of such scar tissue may complicate lymphatic transplant.^18^

Nguyen et al performed meticulous debulking of upper extremity recipient site tissue, with 90% of their patients reporting improvements in lymphedema‐related symptoms and recipient‐site complications limited to hematomas and seromas.^27^, ^28^ An additional advantage of VOLT is that the abundance of lymphatic tissue in the omentum allows for the division of a single flap into two smaller flaps, making double VOLT a promising option.^19^, ^22^ Double VOLT, with a combination distal and middle or proximal and middle inset, was utilized by several authors to take advantage of benefits of each site.^17^, ^19^, ^22^, ^23^ Distal inset allows for capture of gravity‐dependent lymph accumulation in the distal upper extremity, while middle or proximal inset allows for orthotopic transplantation with the goal of replacing the iatrogenically damaged lymphatic tissue.^24^, ^29^ Further research is required to determine the advantages of single vs double VOLT. Similar to donor site selection, recipient site selection may depend on patient indications, patient and surgeon preferences, and complicating factors.

Ciudad et al note that excisional procedures may be necessary to optimize lymphatic drainage following VLNT.^17^ A hallmark of chronic lymphedema is the hypertrophy and fibrosis of adipose tissue, which reduces lymphatic function. While VLNT replaces damaged lymphatic tissue, SAL allows for the removal of diseased tissue, thus improving limb volume and lymphatic load.^30^ Two studies utilizing excisional procedures following VOLT appeared to improve upper limb volume reduction/circumference to a greater degree than the studies utilizing VOLT only.^17^, ^19^ However, this must be researched further to form a reliable conclusion.

While an open surgical approach for VOLT flap harvest has been preferred historically, advances in laparoscopic techniques call for further investigation into the risks and benefits of each method. Manrique et al compared patient outcomes of 126 laparoscopic harvests and 51 open harvest over a 6 year period and found that the laparoscopic cohort experienced less postoperative pain (3 vs 7, on a scale to 10), faster return of gastrointestinal function (1 vs 2 days), shorter hospital stay length (2 vs 5 days), fewer complications, and increased patient satisfaction scores based on donor site pain and scarring.^31^ However, other authors emphasize the importance of laparoscopic and microsurgical experience in VOLT flap harvest, and some advocate for an open approach if the surgeon does not have adequate familiarity with advanced laparoscopy.^21^, ^32^ Kenworthy et al prefer the open approach due to finer control of microsurgical instruments and tissue, limiting potential damage to the flap vasculature.^23^ Only two studies in this review preferentially utilized the open approach, so it is not possible to draw conclusions of either donor site complications or recipient site outcomes in laparoscopic vs open approach from this review. Therefore, this should be an area of further research. Nevertheless, both options are appropriate with the proper training and the decision of which to use is generally surgeon‐dependent.

An emerging technique is robotic harvest of the omental flap. Research on this topic is sparse, but has shown promise. Similar to laparoscopy, the surgeon must be experienced in the application of robotic surgical techniques. The robotic approach presents several potential advantages over laparoscopy for VOLT flap harvest, including three‐dimensional visualization, tremor elimination, and superior instrument articulation.^33^, ^34^ A study of a small cohort of patients whom underwent robotic VOLT flap harvest found that the surgeon was able to dissect the lymphatics and vasculature with greater precision, although the operative times were longer.^35^ Recently, authors successfully performed robotic VOLT for lymphedema using a single‐port robotic platform. This further reduced donor site morbidity and limited the number of incisions necessary for omental harvest.^36^ Early results justify further investigation into the application of robotic surgery for omental flap harvest.

This findings of this review seem to corroborate previous studies and systematic reviews of surgical lymphedema treatment. Specifically, a 2019 report by Forte et al of omental lymph node transfer for both upper and lower lymphedema similarly suggested positive benefit from VOLT in affected patients.^3^ In their report, the authors found that the majority of included studies suggested objective improvement in circumferential reduction and volume reduction, although not all patients reported subjective improvement. We similarly found that VOLT is effective for the treatment of upper extremity lymphedema, and potentially to an even greater degree than previously suspected. Although similar in scope, the present review is differentiated from this and other prior reports due to its specific focus on treatment of upper extremity lymphedema with VOLT. This has allowed for deeper analysis with respect to a comparison of average circumferential reduction and volume reduction between studies. In addition to a more focused question, the current report provides an updated and comprehensive perspective, which includes query and inclusion of additional databases, studies, and patients not analyzed in previous systematic reviews. This point is key, as our understanding of lymphedema and its surgical management are continually changing. Compared to extirpative procedures for lymphedema treatment such as direct excision and liposuction, physiologic approaches such as VOLT are still in their infancy. As with examination of any budding field, it becomes that much more important to provide ongoing analysis of novel findings to ensure that the therapies in question are safe, effective, and appropriate. Therefore, the relative value of an updated review on surgical lymphedema management such as the current report is highlighted.

This review reports and provides analysis for all English‐language original studies from four databases that discuss the management of upper extremity lymphedema using VOLT. There are several limitations to this review, however, including the limited number of studies, relatively small cohort size, variation in surgical technique and protocols, and heterogenous methodology for objective limb measurements. Additionally, the majority of published studies provide descriptive findings rather than a systematic viewpoint. As a result, it is difficult to definitively form conclusions regarding proper patient selection and harvesting technique with the available evidence. Ongoing and more comprehensive studies will enable stronger conclusions moving forward. Next, while VOLT appears to have great promise in the treatment of upper extremity lymphedema, the relatively short follow‐up periods in the studies make it difficult to extrapolate long‐term safety and efficacy of this intervention. Lastly, several of the studies in this review tested VOLT as a treatment for both upper and lower extremity lymphedema, but were included due to reporting the data for upper extremity separately from the data for lower extremity. However, a small number of studies were excluded due to data not being separated. Therefore, pertinent upper extremity data from these excluded studies could not be obtained and included in this review. With these limitations in mind, VOLT still holds great promise as a treatment for upper extremity lymphedema, and warrants further investigation into the long‐term effects and ideal surgical protocol.

## 5 CONCLUSION

VOLT is effective and safe in the surgical treatment of UE lymphedema. This review found that VOLT facilitates a reduction in limb volume/circumference, subjective improvement, and is associated with relatively few complications. Ongoing research is necessary to characterize indications, limitations, efficacy, and patient satisfaction of VOLT for UE lymphedema.

## CONFLICT OF INTEREST

The authors report no conflicts of interest with respect to this work.

## ETHICAL STATEMENT

Not applicable.

## AUTHORS' CONTRIBUTIONS

All authors had full access to the data in the study and take responsibility for the integrity of the data and the accuracy of the data analysis. *Conceptualization*, N.R.J, A.M.R., C.M.T.; *Methodology*, N.R.J., C.M.T.; *Investigation*, N.R.J., R.A.T., C.M.T.; *Formal Analysis*, J.N.R., A.J.F., A.M.R., C.M.T.; *Resources*, R.A.T., F.R.A., A.J.F., A.M.R.; *Writing ‐ Original Draft*, N.R.J., C.M.T.; *Writing ‐ Review & Editing*, R.A.T., F.R.A., A.J.F., A.M.R., C.M.T.; *Visualization*, R.A.T., F.R.A., A.J.F., A.M.R.; *Supervision*, C.M.T.; *Data Curation*, N.R.J., R.A.T., F.R.A, C.M.T.; *Software*, R.A.T., F.R.A., A.J.F., A.M.R.; *Validation*, A.M.R., C.M.T.; *Project Administration*, C.M.T.

## Data Availability

The data that support the findings of this study are available from the corresponding author upon reasonable request.
